# Manual of Surgery, for Use at His Lectures

**Published:** 1845-07

**Authors:** 


### Art. VIII.

1. *Handbuch der Chirurgie, zum Gebrauche bei seinen Vorlesungen.* Von Maximilian Joseph Chelius, der Medicin und Chirurgie Doctor, &c. Erster Band. Sechste, vermehrte und verbisserte original-auflage.—*Heidelberg und Leipzig,* 1843.

*Manual of Surgery, for use at his Lectures.* By M. J. Chelius, Doctor in Medicine and Surgery, &c. First Volume of the Sixth, enlarged, and improved edition.—*Heidelberg and Leipzic,* 1843, pp. 1012.

2. *A System of Surgery.* By J. M. Chelius. *Translated from the German, and accompanied with additional Notes and Observations,* by John F. South, Surgeon to St. Thomas's Hospital. Part I. 8vo, pp. 112.

The test of time and often repeated editions have stamped the Manual of Professor Chelius with an acknowledged value, which all who have made themselves acquainted with its contents will readily concede to it.

In bringing the present volume before the notice of our readers, it is our intention to present them with a brief analysis of the general plan of the work, and of some parts of its contents; and to illustrate the same by occasional reference to the text; more than this will be unnecessary, as an early opportunity will probably be given to the public of a closer intimacy with the complete work in an English garb.

The scope of Professor Chelius' Manual is indicated by its title; it professes to treat, systematically, of the science and art of surgery, but within such compass as to render the work an appropriate introduction and companion to his lectures. The care, however, which has been bestowed upon its construction, and the labour which its research evinces, would be ill-repaid were it confined to this sphere; and we may conscientiously say that we know of no Manual of Surgery, on the whole, more deserving of public confidence, or more valuable as a guide and refresher to the young practitioner.

The work is opened by a short introduction on the objects and aim of surgery, and its relation to other branches of the healing art; together with the author's estimate of the natural and acquired qualifications of a surgeon. That we are not opposed to the diffusion of information, and elevation of the general standard of medical education, we trust our pages will bear ample testimony; but we do not profess ourselves to belong to that class who desire to see all professional distinctions extinguished; and we are therefore gratified to find a master in our noble art recognising an elevated standard as essential to surgical proficiency.

"The study" says the Professor, "and the practice of surgery are surrounded by difficulties of no ordinary character. The dexterity and safety with which surgical operations must be performed can only be acquired by long practice on the dead; the opportunities for this are few, and still more rarely do we meet with the perseverance necessary to overcome all the disagreeables which are associated with it. How many have reason to regret their neglect of practising on the dead, when called on to operate on the living! In many cases the life of the patient is each moment in the hands of the operator; the struggles of the sufferer, his cries, and a peculiar feeling to which no surgeon, especially in the commencement of his career, is a stranger, shakes his necessary equanimity, and makes him anxious and unfit to complete his work with celerity and safety. Nor need we wonder when we peruse the candid acknowledgment of the great Haller: 'although I have filled the chair of surgery for seventeen years, and have frequently exhibited the most difficult operations in surgery on the dead, yet have I never ventured to use my knife to the living, lest I should do mischief.'" (p. 13.)

We have not been tempted to make the above quotation by any novelty in the sentiments it contains, but to give force to our opinion that surgical operations are in the present day too lightly esteemed, and frequently practised by those whose opportunities do not justify them in thus tampering with human life. It is impossible that all can be good operators, even were the natural qualifications of all on a par; and we do not hesitate to deprecate, and denounce as unjustifiable and cruel, (to use no stronger term,) the eagerness with which an occasional operation of importance is sought and undertaken for the sake of professional celebrity; and without the excuse, which might have been pleaded before the establishment of the numerous county hospitals, of distance from the metropolis.

Before presenting his readers with his own classification of disease, our author has to dispose of the often disputed question of the boundary line between that which falls to the care of the physician, and that which it is the surgeon's duty to treat. The merits of this *questio vexata* we do not intend now to discuss; but satisfy ourselves with giving in our adhesion to the Professor's opinion, that any attempt at classification according to the internal or external seat of the disease, or that which is drawn exclusively from the nature of the treatment required (whether mechanical or medicinal,) is altogether unmeaning and absurd, and to be rejected as such. Custom has to a certain extent fixed the limit, which, where it is clearly understood, should be respected; but, at the same time, we think with Professor Chelius, that it is desirable "to seek for some general character of disease, which may serve in some measure to establish a nosological division, and to indicate those diseases which may appropriately be denominated surgical." Our author proceeds to observe that, as the phenomena of life afford us the means of defining a healthy condition as a state of harmony between the physical and organic functions, so the predominance of the one or the other, constituting a deviation from the normal type, presents us with a division of disease into 'dynamic' and 'organic;' and that "organic diseases are especially those which have their origin in a lesion of the natural quality, form, and structure of the organic frame, and may be produced by disturbance of the mutual dependence of organs, by the abnormal union of parts, by the presence of foreign bodies, by the degeneration of organic structures or production of new material, and lastly by the absolute loss or supernumerary presence of organs." The conclusion and inference from these remarks is, that surgery is entitled to all those organic diseases "which have their seat in such parts as are accessible to the sense of touch, or require the employment of mechanical means for their cure." (p. 15.)

The division and classification of disease derived from the above foundation is as follows: 1, of general and specific forms of inflammation, and of inflammation affecting particular organs; 2, of diseases which consist in the disturbance of the physical relation of parts, including wounds, fractures, dislocations; chronic lesions, abscesses, scrofulous and venereal affections, diseases of the vascular system, &c. &c.; 3, diseases which are characterized by the abnormal cohesion of parts; such as stiff joints, adhesion of the gums and cheeks, of the sides of the vagina, &c.; 4, of foreign bodies, whether introduced into the system, or generated and retained there: this head includes the presence of foreign bodies in the trachea and œsophagus, and retention of the fœtus in utero, or urine in the bladder, hydrocele, &c.; 5, this section comprehends all diseases which are typifyed by hypertrophy or degeneration of organized structures; and the two following treat of the loss of organs, and their supernumerary presence; whilst the eighth and last section is devoted to surgical operations in general and in particular.

It is not our intention to comment on the above arrangement, which, in common with all classifications, possesses its disadvantages as well as its merits; though in one sense simple, it is certainly rather confusing in another; as we find diseases, which possess nothing in common beyond the very general type to which they are referred, mingled together. We shall also pass over in silence the first general section on inflammation, which contains nothing new, and is a little antiquated in some of its views: but it is much, now-a-day, to expect the practical and practising surgeon to be quite conversant with all the changes and revolutions which our present extended means of observation are progressively effecting. We shall therefore proceed to select indiscriminately from different sections of the volume, and will commence with that on "Inflammation affecting joints."

*Inflammation of the synovial membrane and of the cartilage of joints* is thus contrasted:

"In the commencement (of synovitis) there is slight pain, which either involves the whole articulation, or is confined to one point. Occasionally, however, when this is very severe, fever is also present, and the motion of the joint becomes impracticable. After a time swelling commences, and fluctuation becomes perceptible, the locality varying according to the form, but most distinct where the soft parts offer least resistance. If the attack of inflammation last longer or be often repeated, the swelling waxes larger and firmer by the breaking up and degeneration of the synovial membrane and ligaments; severe pain ensues, accompanied by loss of sleep and hectic fever; the swelling yields at various points, and the power of the patient is gradually exhausted. In the most favorable cases anchylosis is the result." (p. 129.) . . . . "The cartilage of joints may be the primary seat of inflammation and ulceration, which subsequently spreads to the other constituents of the articulation. In the beginning the patient is sensible of slight and transient pain, which is augmented by motion of the joint, and diminished when it is at rest. Gradually this symptom becomes more persistent, and extends from the joint over the bone. Many weeks or even months elapse before any swelling of the articulation is apparent, and when it becomes so it is accompanied by a slight external blush of inflammation. But this swelling is neither marked nor fluctuating, and the generel form of the articulation is preserved. Sooner or later matter is formed in the joint, which discharges itself, and the patient sinks with symptoms of hectic. Although the early stage of this disease is almost always insidious, yet the occurrence of peculiar circumstances may aggravate the attacks where the disease is disposed to assume an acute form." (pp. 131-2.)

We thus perceive, what indeed Professor Chelius afterwards remarks, that his observations agree with those of Brodie, and in opposition to the opinions of others, that the latter affection is distinct from that of inflammation affecting the synovial membrane or extremities of the long bones; at the same time he admits that the cartilage "may not be invariably the primary seat of disease in these cases, but that *partial* inflammation may be set up in the synovial membrane, or first exhibit itself in the cancellous structure beneath the cartilage." In the treatment of these affections our author's opinions agree with those generally received and adopted in this country; active depletion in the earlier stage, and counter-irritation when the disease has passed into a chronic form. In praising issues, he justly remarks that good is not to be looked for as an immediate result of their employment; but that free suppuration from the surface must be maintained for a long time, whilst rest is strictly enjoined, before the wished-for effect is obtained. The onset of inflammation of the spongy texture which constitutes the extremities of long bones is described as similar to that of inflammation of cartilage; but the character of the elastic swelling, "which derives its form from the distended head of the bone, and varies in dimensions according to the tension or rest of the joint," is noticed as the chief point of contrast, and as characterising the affection of the osseous structure itself.

*Disease of the hip-joint.* The section on 'Hip-joint disease' is well drawn up, and the descriptions are graphic and natural. This troublesome affection is divided into three distinct stages; commencing with the slight pain and occasional stiffness, which is gradually merged in the more marked characteristics of lengthening of the diseased limb, shrinking of the buttock, and abnormal position of the great trochanter, which constitute the second stage; whereas the shortened extremity indicates the further progress of the disease, which peculiarly characterizes the third and last stage. Our space will not allow us to enter at large upon this subject, but we shall make room for one or two remarks. "The shortening of the extremity," says the Professor, "which is usually observable in the first stage of acute inflammation of the hip-joint is always apparent (not real) and is due to the tilting of the pelvis *upwards* on the affected side." In this opinion we entirely coincide, and believe that the natural effort of the patient to relieve the suffering limb is the cause of that which some writers have set down to muscular spasm.

"The lengthening of the extremity, which is observed in the chronic form of hip-disease, or the later stage of the acute, when morbid changes in the joint have already taken place, may be either *apparent* or *real.* The apparent shortening may here also depend on the tilting of the pelvis, as the sufferer in this tedious disease manages still to keep about, (dragging the affected limb,) and thus throws the whole burden of support on the healthy limb; by this means as well as the constant position maintained in bed, the pelvis becomes in such wise twisted as to be elevated on the sound side, and depressed on the opposite. In the later stage of the disease, when the morbid changes in the joint have extended further, actual lengthening of the diseased extremity becomes evident, which does not depend on any mechanical derangement of the relation between the head of the thigh-bone and its socket, and the extrusion of the head of the bone, by reason of its great bulk; but it is due to the extension of the capsular ligament, to the accumulation of fluid, the relaxation of the capsular ligament, and the entire rest of the muscles." (pp. 139-40.)

On the subject of experiments performed by Weber, from which he infers the important influence of atmospheric pressure in preserving the integrity, as regards relation, of the articulating ends of bones, our author remarks that the results obtained by his countrymen are of the most lively importance as bearing upon diseases of the hip-joint. We subjoin his reasoning upon the subject, as we think that scarcely a due value has been attached to the practical bearing of these experiments in this country. It is hardly necessary to remind our readers that, in the experiments alluded to, Professor Weber has shown that after division of all the muscles, and even of the capsular ligament itself (say of the hip-joint,) the weight of the entire limb is insufficient to overcome the resistance to dislocation of the head of the bone; the sequel of the experiment proving the operative cause, viz., the admission of air by a perforation of the acetabulum from within. These experiments are not difficult to perform, and are satisfactory in their results. (See a brief account of them in our Second Volume, p. 236.)

"We have therefore every reason to conclude," says our author, "as we have already pointed out, that the explanation of shortening of the extremity by pressure operating forcibly upon the corresponding surfaces of the articulation is altogether inadmissible; and on the other hand, that relaxation of the muscles alone is quite incompetent to produce lengthening of the limb; and further, that the existence of a certain space between the head of the bone and its socket, which was formerly assumed, and imagined to change with each contraction of the muscles and to be augmented during their relaxation, is altogether ideal; but that, on the contrary, the most perfect and intimate contact must exist. It still further follows that, in cases where a real lengthening of the limb exists, an essential precursor of this symptom invariably is the destruction by morbid changes of the natural contact of the articulating surfaces; and we are thus driven to the conclusion that this condition is only to be explained by attributing it to the *concurrent relaxation and extension of the capsular ligament from accumulation of fluid, and to the relaxation of the muscles.*" (p. 141.)

The characteristics by which hip-disease is distinguished from other affections, such as nervous pain in the hip, congenital luxation, &c., are well treated. Two cases, says Professor Chelius, "have I seen of congenital luxation of one thigh-bone only, which were actually mistaken and treated as hip-disease." If the symptoms were as clearly marked as in a case of this singular congenital affection which we lately witnessed, in which both joints were dislocated, we should judge that the practitioners in question could have been but ill acquainted with the anatomy of the parts in their normal state, or with the ordinary symptoms of hip-disease. Our author's experience as regards the ordinary course of these affections does not differ from that of others who have possessed similar opportunities of witnessing its ravages: "the prognosis," he observes, "is always unfavorable, but especially so when the mischief has its origin in the ligaments or synovial membrane; in the earliest period only is a perfect cure possible." The treatment recommended is simple and sound.

*Tetanus.* In turning to the subject of Tetanus, we find the following observations on its origin, or rather its proximate cause:

"It is most probable that the immediate cause of tetanus is an inflammatory condition of the nervous system; at least post-mortem examinations have proved the existence of inflammation in the nerves and their sheaths, of the spinal cord and brain, with serous effusion between their tunics. The spinal marrow seems to be specially affected; and even where the indications of inflammation are not pronounced, there are evidences of irritation and congestion. I found in one case the most unequivocal signs of inflammation in the medulla spinalis, the structure of which was broken down and reduced to a pulpy condition for the space of an inch." (pp. 237-8.)

We have been less fortunate in our examinations. The remedies enumerated for this intractable affection are, bleeding, the exhibition of mercury in combination with opium or morphine, mercurial infriction and warm bathing. The cold bath is admissible in the earliest stage only; cupping-glasses, along the spine, combined with general bloodletting our author thinks the most applicable treatment where the patient is young and robust, and the spasms severe; more especially if inflammatory fever be present. We quite agree in a further remark upon the value of opium in this affection; and in the feasibility of administering large doses (of several grains) without producing narcotism.

*Wounds of the head.* The section on "wounds in particular" is introduced by a chapter on "Injuries of the Head;" in which the various textures and parts obnoxious to external violence are brought under notice, together with the injuries to which they are severally liable: on the details of this chapter we shall make two or three remarks. Wounds of the textures superficial to the skull our author divides into four classes:

those affecting the scalp alone, or extending to the aponeurosis, muscles, and pericranium. We first have some practical remarks on the disproportioned mischief which is not unfrequently observed as the result of wounds (especially punctured wounds) involving the aponeurosis and pericranium; the history of these cases (most commonly occurring in full-blooded and intemperate individuals) being, that inflammation is set up in the fibrous tissues included in the injury, which runs a rapid course and terminates in suppuration and death of the parts affected. The concomitant symptoms require often active antiphlogistic treatment, though the extent to which this is to be carried requires great discrimination on the part of the practitioner,—at least such is the case in our London hospital practice; and the indiscriminate employment of *cold* lotions to the head is to be deprecated; indeed there are few instances (where lotions are indicated,) in which the *tepid* application is not more agreeable to the patient, whilst it is equally efficacious and attended with less risk. With the closing remarks on the treatment of these injuries we entirely coincide, viz.: "that the safest course to avoid the most dreaded results is to make an early (and free) incision into the swelling, and thus give a ready exit to the collected fluid and dead cellular tissue."

Our author's treatment of "scalping" wounds of the head is, according to our experience (as a general practice) unnecessarily interfering. Sutures ought never to be employed when they can possibly be dispensed with; and the dressing should be as light and unincumbered as possible; charpie, compresses, and bandages are rarely required, and generally mischievous. Again, in the more serious question of the use of the trephine, we cannot agree in the general principles which are to guide the practitioner; and we are rather surprised that the experience of so cautious a surgeon should have conducted him to such results. The following are some of the Professor's remarks upon the subject:

"Fractures of the skull with depression call for the employment of the trephine on the spot, even in the absence of any symptom of compression or irritation of the brain. Such symptoms declare themselves sooner or later, and then the operation will be no longer of any avail. . . . . When the condition of the wound is such as to admit of a ready removal of the splinters or shattered fragments of bone, and of a free escape for the extravasation, then only may trephining be regarded as superfluous. . . . . . By very great violence the sutures of the skull may be separated. If the surgeon has satisfied himself by extension of the wound and the requisite incisions that such is the case, and that the separation in question is not sufficient to permit a ready exit to the secretions from both sides, he should trephine. . . . . If injuries to the skull (produced by great external force, such as fire-arms, &c.,) are attended with splintering of the bone, if the diploe is pressed upon, the outer table fractured, and the inner splintered, or where there is depression of fragments of bone, the trephine must be immediately had recourse to." (pp. 249 et seq.)

We shall make one more quotation on this subject, from the chapter on "Inflammation of the brain." After describing the symptoms and treatment of this condition in its earlier stages, Professor Chelius remarks:

"When the symptoms of suppuration (of the brain) have become evident, the prognosis is very bad; and the only chance of rescuing the patient is the speedy employment of the trephine on the spot where the pain or inflammation was first complained of, and in so doing, if the suppuration have extended itself, it will usually be found necessary to apply the trephine at several points, and to divide the dura mater, and cut into the surface of the brain itself, when the position of the collected matter calls for it." (p. 256.)

The Professor justly adds that, where the pus is diffused, either over the surface or between the dura mater and skull, the operation will prove entirely useless; and that even when the dura mater is divided it is by no means easy to diagnosticate the exact locality of the abscess which is supposed to exist in the brain. It is not our intention to comment on the practice advocated in the above quotations, but merely to remark that it differs importantly from our own modern practice in England.

*Fractures.* We shall lastly select the section on "Fractures in general" for notice, and with this close our present brief review. After enumerating the different forms of fracture to which bones are obnoxious, our author proceeds to comment on the relative liability to these injuries at different ages and under different circumstances. The occurrence of fracture from comparatively slight causes in the old, and under different predisposing forms of disease, he attributes to the disproportion which the process of deposit holds to that of absorption, by which the resistance of the texture is weakened. On this subject our author subsequently avails himself of his French translator's note, from which we also extract the following observations:

"The compact tissue of the shafts of long bones is closer and more solid in the old than in the growing; it rings when struck, and is hard and difficult to break. It is true that the caliber of the medullary canal is augmented, but this is exclusively at the expense of the inner spongy laminæ, which have nearly altogether disappeared. But near the extremities of the cylindrical bones an increased absorption may be remarked, by which the substance of the bones at these parts loses in thickness; the spongy tissue presents larger cancelli, especially in the neighbourhood of the medullary canal, which thus appears continued into the extremity of the long bones. This difference indicates why the porosity of the ends of cylindrical bones in the aged has more influence on their fragility than the lack of elasticity in their shafts, which is moreover counterbalanced by their greater compactness. Thence it arises that fracture of the extremities of the cylindrical bones in the old, (especially of the neck of the femur,) is very frequent, whilst that of the shaft is even more rare than in the growing." (p. 345.)

The following are enumerated as the symptoms by which the existence of fracture may be determined: by the altered form and direction of the limb; by its length, thickness, and straight bearing, as well as by the disturbed function, unnatural mobility and crepitation of the injured member. In connexion with this last means of *diagnosis* our author notices Lisfranc's recommendation that the aid of the stethoscope should be called in, (of course he presupposes the ability of the surgeon to use it;) but dryly adds that when crepitus is present, it may be always as readily detected without the aid of this instrument: we may ourselves add that, if the sense of touch fail to distinguish between the crepitus of fracture and of effusion, it will be vain to seek a solution of the difficulty by the aid of the ear. The *prognosis* in cases of severe fracture, as determining the course of practice to be pursued, often involves the alternative of loss of life or limb; and it is therefore of the utmost importance to bring all the information we can collect to bear upon this momentous question. Amongst the several points enumerated by Professor Chelius as influencing the prognosis, he mentions the behaviour (Betragen) of the patient during the cure. Whether he means to include under this head the general *moral* tone and temper of the sufferer we do not clearly understand; but certain we are that this ought to have a very great weight with the practitioner, and may even be of sufficient importance alone (other points being equally balanced) to determine him in sacrificing or saving a limb. It has repeatedly occurred to us to witness in hospital practice the most frightful injuries recovered from, with scarcely the interruption of a bad symptom, where the temper and general *morale* of the patient is equal and calm, whilst the converse holds equally true: this is also especially remarkable after severe operations. In the directions that follow on the subject of "extension, counter-extension, coaptation," and the mode of putting up fractures, our author puts us in mind of the formal directions given by some of our older authorities; and we cannot quite agree with him in some of the details of his practice. The employment of a compress over the seat of fracture is in our opinion rarely useful and often mischievous: and again, the direction to place the limb in a straight position when the fracture extends through the middle of the cylindrical bones (p. 352) is by no means to be acted on as a general rule; at least such is the result of our own experience. A little further on active antiphlogistic treatment is spoken of (bloodletting, leeches, &c.) as occasionally required after severe fracture; this may be admissible or even requisite, though very rarely, in the full-blooded healthy countryman, but the exceptions are extremely rare when such practice could be adopted without extreme risk in London practice. In speaking of wounded blood-vessels associated with fracture, our author recommends the practice of cutting down upon them and tying them; or where that is impracticable, he adds, that the trunk of the artery must be sought and tied above the seat of injury, (p. 355.) This will be found more difficult in practice and dangerous in its results than it might appear in theory, and can rarely be acted on: the limb must generally be sacrificed in time to save the patient from the death which awaits him either from secondary hemorrhage, or gangrene consequent on deficient supply of blood. Professor Chelius seems fully alive to the value of opium in the treatment of nervous delirium following severe injuries to the bones; and also admits the propriety of not cutting off all supply of stimulus from those who have been accustomed to it: this last often proves our sheet anchor in the treatment of these troublesome cases, where a tendency to nervous delirium exhibits itself soon after the accident. The conclusion of the present section is occupied with the treatment of badly-united fracture, by the operation of re-breaking them; we cannot say that our personal experience affords us any commentary upon this practice; we therefore only quote our author's observations which are derived principally from Oesterlen's experiments, which were published some years since.

"Where the union of a fractured bone is so bad as to materially curtail its usefulness and be a source of suffering to the patient; and when extension and bandaging are no longer of any avail, and the patient is neither too old nor weak, nor the subject of phthisis, gout, &c., the bone may be again fractured in the callus with the most happy results. No general rule can be laid down respecting the period to which this operation may extend: it must depend upon the condition of the patient, the nature and seat of the fracture, and the state of the callus.

Oesterlen's observations prove that the operation has succeeded in the thigh and leg after the lapse of seventeen weeks; and some of his experiments on adults succeeded as late as the twenty-sixth week. In young subjects, and where the bones are not very strong, as in the forearm, the refracture through the callus has been effected as late as at the end of a year, especially when the callus is not very thick. If the callus be recent and moderate in quantity, the operation in question may be accomplished by the hand alone; otherwise the assistance of a machine constructed for the purpose is required." (p. 359.)

We shall here bring our observations to a close; and in summing up we feel called upon to make a few parting observations for a reason adverted to in the commencement of the present article, and which we deem necessary, as our readers would perhaps find it difficult to gather them from the quotations we have made from the sections which have been selected as best adapted to illustrate the general principles and qualities of the work before us. We cannot help remarking a disproportion in the treatment of the more important and trivial subjects; this observation is, however, more applicable to the detail than to the general handling of the subjects; we should have been better pleased to have seen points of minor importance thrown more into the background, and those of greater moment brought more prominently into relief: i. e. for the sake of the student who has not yet learned to make this distinction for himself. In the same category of faults (for such they are in a *Manual,*) we may notice that it is difficult in some instances to judge of the frequency of the occurrence of certain rare symptoms in a disease, or of the necessity for peculiar or unusual forms of treatment. The local treatment of some injuries, as well as diseases, is unnecessarily encumbered and complex; and many casual remarks prove their author to be *a little* behind his brethren, in Germany as well as in England, both in theory and practice; probably this may arise from the learned Professor being less conversant with men than with books. The reading and research evinced are very great, and render the treatise valuable as a work of reference. The above criticisms are, however, in the main applicable to faults of minor importance; and may (as we doubt not they will) be corrected in the forthcoming English translation. The general character of the work leaves us no room to doubt that Professor Chelius fully merits the high reputation he possesses as a practical teacher and a good surgeon; and the more careful analysis which we have bestowed on the volume before us only leads us to a recapitulation of the opinion with which we commenced our review,—that we can conscientiously recommend the work as a safe guide in practice, and as fully deserving the confidence of the professional public.

Since writing the above we have received the first 'Part' of Mr. South's promised translation of our author. The 'conditions' of publication include the completion of the work in twelve monthly parts, forming two octavo volumes; from this we infer that the annotations (which in bulk considerably exceed the text) will not be quite so extended in the succeeding numbers, or we very much question the practicability of fulfilling this engagement which the editor volunteers to make with the public. The contents of the present part consist of 'Inflammation' generally and specially considered, together with its terminations or proximate consequences: 'Erysipelas' constitutes the second section; and the chapter on 'Burns' is commenced.

It is not our intention at present critically to analyse Mr. South's labours; but we should be guilty of an injustice to him and to our readers if we did not cordially recommend his work as having fair promise of forming, what it is the translator's ambition it should be, a sound and comprehensive system of practical surgery. The notes and text are so intermingled as to render it continuously readable, without presenting those abrupt transitions which are so disageeeable in many works similarly arranged. The faults of omission, &c. at which we have hinted in our comments on the first chapter of our author's work, (viz. that on 'inflammation,') have been amply compensated by the copious and excellent digest of his translator and annotator, who is justly proud of availing himself of the labours of our own countrymen in this department of pathology, whilst he gives their due meed of notice and respect to the contributions of our continental brethren. The references which are given to original works have evidently been carefully collated, and will be found of great value to the student and practitioner, who may wish for more copious information on any particular branch of surgery; and the practical remarks and illustrations with which the work abounds are a good guarantee of the translator's ability to do justice to his task, at the same time that they prove that Mr. South has not failed to avail himself industriously of the large opportunities which his hospital appointment has afforded him. We could have wished that the translation had been rather less literal and cramped in style, which might have been done without any sacrifice, and with very little additional trouble: we should then have been spared the frequent repetition of the expression 'and so on' (und so weiter), together with other Germanisms which do not sound well in English. We would also recommend that some self-denial be exercised in the introduction of cases; and that those which are inserted be as much abridged as is compatible with their application to the subject which they are designed to elucidate. On the whole, Professor Chelius has, we think, ample reason to be satisfied, thus far, with the English garb and deckings of his offspring; and, for our part, we are only surprised that a surgeon with the resources which Mr. South appears to possess, should not have preferred the paternity of an original work, to the adoption of that of another. But such are the peculiarities of different minds, that some men can best mould from the rough clay, whilst others prefer working up into their own taste and fashion the model which has been prepared for them.

As the present work is to appear periodically, we may take the opportunity of expressing a hope that Mr. South will redeem that part of his pledge, by which he engages that a number shall appear on the first of every month; and thus set an example to other editors and authors, the conduct of many of whom, in this respect, cannot be too strongly reprobated. To inveigle the public by the bait of fair promises, and then, when fairly hooked, to neglect them, play with them, or fairly turn them adrift, is a species of literary delinquency with which we have no patience; but we expect better things of Mr. South.

